# Transgenic overexpression of microRNA-30d in pancreatic beta-cells progressively regulates beta-cell function and identity

**DOI:** 10.1038/s41598-022-16174-7

**Published:** 2022-07-13

**Authors:** Yiping Mao, Jacob Schoenborn, Zhihong Wang, Xinqian Chen, Katy Matson, Ramkumar Mohan, Shungang Zhang, Xiaohu Tang, Anoop Arunagiri, Peter Arvan, Xiaoqing Tang

**Affiliations:** 1 Department of Biological Sciences, Michigan Technological University, Houghton, MI 49931 USA; 2 Department of Metabolism Endocrinology and Diabetes, University of Michigan, Ann Arbor, MI 48109 USA

**Keywords:** Type 2 diabetes, miRNAs

## Abstract

Abnormal microRNA functions are closely associated with pancreatic β-cell loss and dysfunction in type 2 diabetes. Dysregulation of miR-30d has been reported in the individuals with diabetes. To study how miR-30d affects pancreatic β-cell functions, we generated two transgenic mouse lines that specifically overexpressed miR-30d in β-cells at distinct low and high levels. Transgenic overexpressed miR-30d systemically affected β-cell function. Elevated miR-30d at low-level (TgL, 2-fold) had mild effects on signaling pathways and displayed no significant changes to metabolic homeostasis. In contrast, transgenic mice with high-level of miR-30d expression (TgH, 12-fold) exhibited significant diet-induced hyperglycemia and β-cell dysfunction. In addition, loss of β-cell identity was invariably accompanied with increased insulin/glucagon-double positive bihormonal cells and excess plasma glucagon levels. The transcriptomic analysis revealed that miR-30d overexpression inhibited β-cell-enriched gene expression and induced α-cell-enriched gene expression. These findings implicate that an appropriate miR-30d level is essential in maintaining normal β-cell identity and function.

## Introduction

A progressive loss or dysfunction of pancreatic β-cells occurs in the development of type 2 diabetes^1, 2^. Loss of β-cell mass has been ascribed to increased β-cell apoptosis in patients with diabetes^3^. Alternatively, decreased β-cell mass may reflect insufficient expansion of β-cell mass or a failure to adaptively regenerate β-cells in diabetes. Notably, growing evidence supports that a new mechanism underlying β-cell loss is β-cell dedifferentiation, in which β-cells lose their differentiated identity and de-differentiate into bihormonal cells co-expressing insulin and glucagon in individuals with diabetes^4–7^. Studies have shown that these bihormonal cells are present in humans with type 2 diabetes and diabetic mice^4, 8, 9^. Moreover, dedifferentiated β-cells lack the expression of crucial factors that are normally expressed in the mature β-cells (e.g., Nkx6.1, Nkx2.2, Pdx1, MafA, Glut2/Slc2a2) and re-express genes that are solely expressed in α-cells (e.g., MafB, Irx1 and Irx2)^4^. β-cell deficiency is induced by many stress conditions including oxidative stress, endoplasmic reticulum (ER) stress, inflammation and hypoxia^6, 9–12^. However, many aspects of the signaling effectors involved in β-cell loss as well as their underlying mechanisms remain to be explored^13^.

MicroRNAs (miRNAs) are endogenous, short non-coding RNAs that regulate gene expression by suppressing mRNA translation and reducing mRNA stability^14^. Many dysregulated miRNAs have been reported in patients with diabetes and diabetic animal models^15–19^. How these miRNAs systemically control β-cell development and function has only recently begun to emerge with the analysis of miRNA knockout or transgenic overexpressed mice^20, 21^. For example, miR-7 overexpression in β-cells induces β-cell dedifferentiation by inhibiting the transcription factor paired box 6 (Pax6), which further inhibits insulin biosynthesis and β-cell transcription factors including Pdx1, Nkx6.1 and MafA^16^. In contrast, mice with miR-375 deletion exhibit hyperglycemia due to decreased β-cell and increased α-cell mass and function^22^. MiR-483 deletion induces β-cell dedifferentiation in mice fed high-fat diet by elevating Aldh1a3, a dedifferentiation marker^23^. However, the physiological importance of individual miRNAs in regulating glucose homeostasis and diabetes pathogenesis remain largely unclear due to mild phenotypic effects in most of miRNA knockouts.

MiR-30 family contains five members (including miR-30a, miR-30b, miR-30c, miR-30d, and miR-30e) located on three different chromosomes. MiR-30 family members, particularly miR-30a and miR-30d, are highly enriched in human islets and downregulated in islets isolated from individuals with diabetes or diabetic *db/db* mice^24, 25^. Considering that miR-30 family has multiple family members and they may act in a functionally redundant manner, genetic inactivation of single miR-30 member in mice may have no obvious phenotypic consequences. Therefore, in this study, we took a gain of function approach and generated transgenic mice in which miR-30d was specifically overexpressed in β-cells at low and high persistent levels. Whereas transgenic mice overexpressing low-level of miR-30d (TgL, 2-fold) had no obvious phenotypic consequence, those with high-level of miR-30d (TgH, 12-fold) displayed hyperglycemia and β-cell loss following high-fat diet (HFD) treatment. Most importantly, miR-30d overexpression induced downregulation of β-cell-enriched genes and upregulation of α-cell-enriched genes, leading to the presence of insulin/glucagon bihormonal cells in TgH mice. These results, together with our previous report, suggested that abnormal miR-30d impaired glucose metabolism and an appropriate level of miR-30d plays an essential role in maintaining β-cell function and β-cell identity.

## Materials and methods

### Generation of transgenic mice overexpressing miR-30d

The animal experiments were performed in accordance with the protocol (L0219) approved by the Animal Care Committee at the Michigan Technological University. All animal work was conducted following the relevant regulations and guidelines. All reporting data in this manuscript follow the Animal Research: Reporting of In Vivo Experiments (ARRIVE) guideline recommendations^26^.

**Figure 1:**
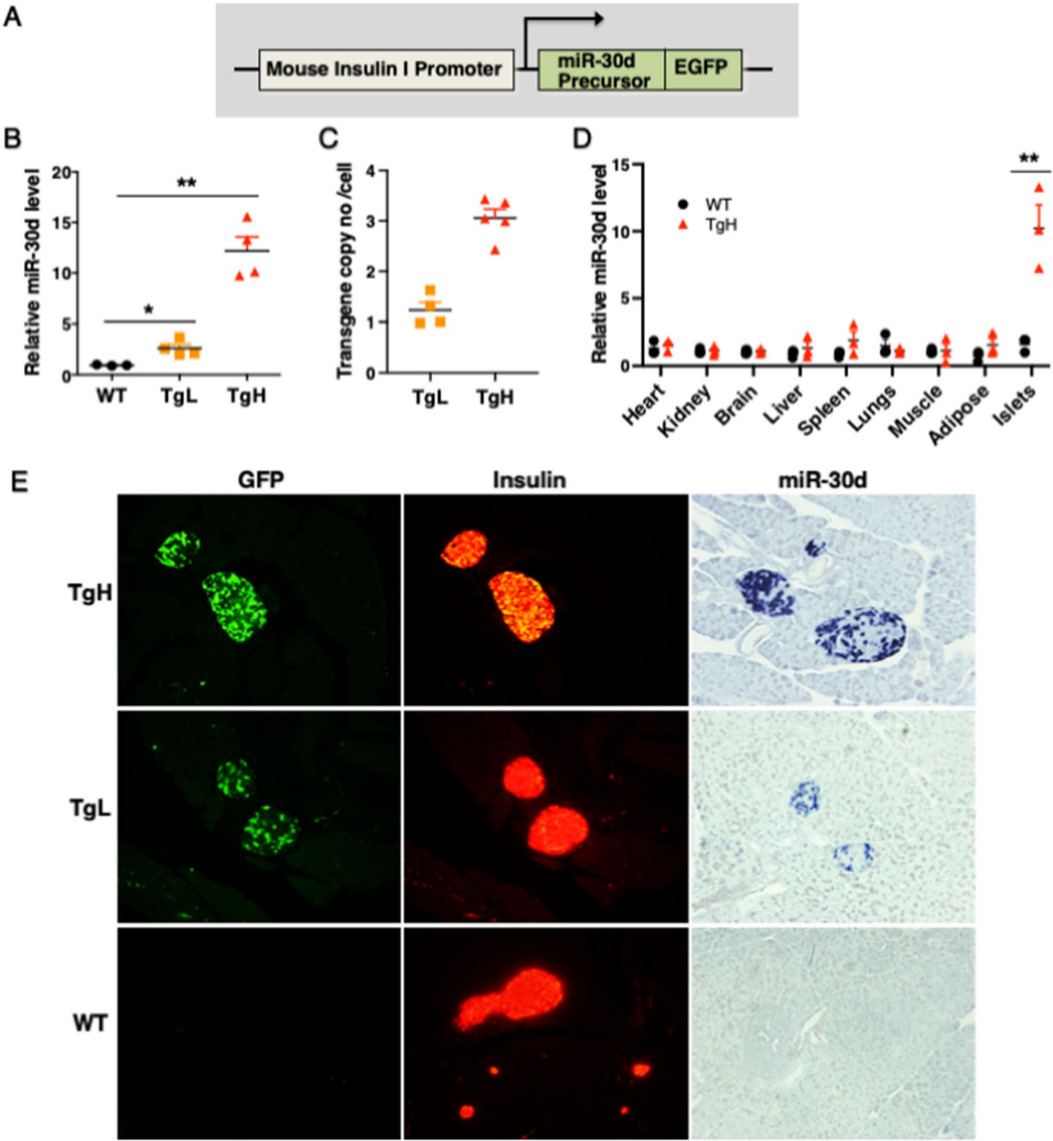
Generation of miR-30d transgenic mice. (**A**) The primary miR-30d fragment was inserted into a β-globin intron associated with an enhanced GFP reporter driven by mouse insulin I gene promoter. The fragment MIP-miR30d-GFP was microinjected into the fertilized eggs of mixed C57BL/6 and SJL mice. (**B**) Two transgenic lines with low and high expression levels of miR-30d (designated TgL and TgH) were selected. RNA was isolated from islets of 10 weeks old male mice (n = 3–4 per group). (**C**) Transgene copy numbers in TgL and TgH lines. The copy number of integrated GFP transgenes was determined on genomic DNA by qPCR using primers for the GFP reporter gene (n = 4–5 per group). (**D**) RT-qPCR confirmed that miR-30d expression was only significantly increased in the islets of transgenic mice. RNA was harvested from islets and various tissues of 14 weeks old male mice (n = 3 per group). (**E**) A representative section of pancreas from 8-week-old mice was visualized by regular immunofluorescence microscopy after staining with anti-GFP (green) and anti-insulin (red) antibodies (20X). In situ hybridization with a DIG-labeled miR-30d probe (blue) confirmed miR-30d is specifically overexpressed in pancreatic β-cells of transgenic mice (20X). All data were presented as mean ± SEM. *p < 0.05, **p < 0.01 versus WT.

Transgenic miR-30d mice were constitutively overexpressed under the transcriptional control of mouse insulin I gene promoter (MIP). The full-length pre-miR-30d fragment was inserted into a MIP-GFP vector provided by Dr. Manami Hara (Fig. 1A)^27^. The linearized fragment was microinjected into the fertilized eggs of mixed C57BL/6 and SJL mice by the Transgenic Animal Core Facility of the University of Michigan. Seven independently derived founders were obtained by PCR genotyping. Two transgenic lines exhibiting low (twofold) and high (12-fold) expression levels of miR-30d (TgL and TgH) were selected and backcrossed to C57BL/6J to generate mice for experiments. The aged-matched non-transgenic littermates were routinely used as control (wild-type, WT) mice. Mouse genotyping was performed by PCR using genomic DNA isolated from tail biopsy and mouse Igf2 gene was amplified in the same PCR reaction as an internal control. The transgene copy number was determined by a quantitative real-time PCR (qPCR) on genomic DNA as described previously^28^. The same primers were used for genotyping and transgene copy number detection as listed in Table 1.

**Table 1:** Primer sequences used for mouse genotyping and transgene copy number determination.

Mice	Primers		Product size (bp)
Transgenic mice (GFP)	Forward	CCTGAAGTTCATCTGCACCA	196
	Reverse	GGTCTTGTAGTTGCCGTCGT	
Wild-type (Igf2)	Forward	TGGATGACTATCCTTGCTGG	280
	Reverse	GAGGCACCCAAAAACCACTCC	

All mice were housed in a pathogen-free animal facility, maintained on a 12-h light/12-h dark cycle and had free access to either regular chow diet or high-fat diet (HFD) containing 60% kCal fat (D12492 from Research Diets) after weaning (4 weeks). Islets were isolated and purified by intra-ductal perfusion of collagenase V (0.5 mg/ml) (Sigma) following the protocol described^29^. Isolated islets were cultured overnight in RPMI 1640 media supplemented with 10% fetal bovine serum (FBS) and 1% penicillin–streptomycin (Thermo Fisher) at 37 °C with 5% CO2*.*

### Plasma insulin and glucagon measurements, and pancreatic hormone contents

Body weight and blood glucose level were measured weekly. Mice were fasted for 6 h and fasting blood glucose was measured from tail vein blood with an Accuchek glucometer (Abbott Diabetes Care) as described^23^. For plasma hormone assay, blood was harvested from orbital venous sinus of fasted mice and measured by Ultrasensitive insulin ELISA kit and glucagon ELISA kit, respectively (Crystal Chem). For total pancreatic hormone contents, whole pancreas was harvested and homogenized in cold acid–ethanol (1.5% HCl in 70% EtOH) overnight at − 20 °C^30^. Pancreatic insulin and glucagon content were then measured by insulin and glucagon ELISA kits (Mercodia), respectively and normalized by pancreatic weight.

### Glucose tolerance test

Glucose tolerance tests were performed in mice between 14 and 18 weeks old as described^31^. Mice were fasted for 16 h and intraperitoneally injected with glucose at 1.0 g/kg body weight. Blood glucose measurements were taken from the tail vein at 0, 15, 30, 45, 60, 90 and 120 min after injection. Blood glucose levels were plotted against time, and the area under the curve was calculated. To measure plasma insulin during glucose tolerance tests, blood samples were collected from the postorbital vein at 0, 15, 30 and 45 min after glucose injection.

### RT-qPCR for miRNA and mRNA

Total RNA from islets was extracted using miRNeasy kit according to the manufacturer's instructions and treated with DNase (Qiagen). TaqMan miRNA qPCR detection system (Thermo Fisher) was used for quantification of miR-30d and its expression was normalized to the relative expression of control snoRNA202^25^. For mRNA quantification, cDNAs were generated using High-Capacity cDNA Reverse Transcription Kit (Thermo Fisher) and quantitative PCR (RT-qPCR) were performed using TaqMan Universal PCR Master Mix (Thermo Fisher) on a StepOnePlus™ System (Applied biosystem). mRNA expression was determined using relative comparison method (∆∆Ct), with Hypoxanthine guanine phosphoribosyl transferase (Hprt) mRNA as an internal standard. The TaqMan assay probes used in the study were listed in Table 2.

**Table 2:** List of TaqMan assay probes for analyzing miRNA and gene expression by RT-qPCR.

miRNA	TaqMan Probe ID	miRNA	TaqMan Probe ID
miR-30d-5p	478606_mir	snoRNA202	001232

Gene	TaqMan Probe ID	Gene	TaqMan Probe ID
Ins2	Mm00731595_gH	Ucn3	Mm00453206_s1
Gcg	Mm00801714_m1	Glut2	Mm00446229_m1
Pdx1	Mm00435565_m1	Irx1	Mm01352526_m1
MafA	Mm00845206_s1	Irx2	Mm01340315_m1
Nkx6.1	Mm00454961_m1	Nkx2.2	Mm00839794_m1
Hprt	Mm03024075_m1		

### Immunoblot analysis

Hand-picked islets were washed once with PBS and then resuspended in RIPA lysis buffer containing protease inhibitors and phosphatase inhibitors cocktails (Sigma) as described^32^. Islets were lysed by repeated pipetting or triturating through a 30G syringe needle. Lysates were resolved on a 4–12% NuPAGE Bis-Tris gel, and then electrotransferred to nitrocellulose membrane for western blotting. Antibodies used were mouse anti-proinsulin (ALPCO), guinea pig anti-insulin (Covance), and mouse anti-vinculin (Millipore). Horseradish peroxidase-conjugated secondary antibodies were from Jackson ImmunoResearch Laboratories, Inc., with protein visualized by ECL (Millipore).

### *In-situ* hybridization and immunohistochemistry

Pancreas was dissected, fixed in 4% freshly prepared paraformaldehyde (pH 7.4) for 24 h at 4 °C, and then processed routinely for paraffin embedding^23^. For miRNA *in-situ* hybridization, sections were first deparaffinized and rehydrated, then treated with Proteinase K (Roch, 40 µg/ml) as described^25^. Briefly, a total of 3 pmol of DIG-labeled Locked Nucleic Acid (LNA) probe for miR-30d (Exiqon) were mixed with 200 µl of hybridization buffer and applied onto the slides in order to hybridize at 37 °C overnight. Slides were then washed using 2X SSC (saline-sodium citrate) solution and incubated with alkaline phosphatase-conjugated sheep anti-DIG antibody (Roche) at 4 °C overnight. Alkaline phosphatase reaction was carried out with 50 mg/ml of nitro blue tetrazolium/5-bromo-4-chloro-3-indolyl phosphate (NBT/BCIP) staining solution at room temperature overnight.

For immunohistochemistry, sections were immunostained with anti-insulin (Sigma), anti-glucagon (Sigma), or anti-GFP (Cell signaling) for overnight incubation at 4 °C. The immunodetection was processed with Alexa Fluor 488- or Alexa Fluor 596-conjugated secondary antibodies (Invitrogen) for 2 h at room temperature. Slides were then mounted with anti-fading mounting medium (Vector Labs) and the images were captured on Olympus FluoView FV1000 confocal microscopy or Leica fluorescence microscopy.

### β-Cell proliferation, apoptosis and β-Cell mass

β-Cell proliferation was determined by 5-bromo-2′ deoxyuridine (BrdU) incorporation as described^31^. Mice were injected intraperitoneally with BrdU (100 μg/g body weight, Sigma) on seven consecutive days. Sections were stained with anti-BrdU (Sigma) and subsequently with anti-insulin. The percentage of BrdU-positive β-cells was calculated and divided by total number of insulin positive cells. β-Cell mass was calculated by multiplying the ratio of insulin-positive areas by pancreatic weight. Islet size was calculated by dividing the area of insulin-positive islets by the total islet numbers. β-Cells undergoing apoptosis were detected by DNA fragmentation using terminal-deoxynucleotidyl-transferase-mediated dUTP-nick-end labeling (TUNEL) assay utilizing In Situ Apoptosis Detection Kit (Roche). At least 3 animals and 5 sections (250 μm apart) per animal were analyzed for all immunohistochemistry studies.

### RNA-seq analysis and pathway enrichment analysis

Total RNA was extracted from islets isolated from wildtype and transgenic mice (3 mice in each group) using miRNeasy kit (Qiagen) with on-column DNase treatment. RNA quality control analyzed on an Agilent 2100 Bioanalyzer. Only samples with an RNA Integrity Number > 9.0 were used for library preparation. cDNA libraries were generated using a stranded RNA-seq library preparation kit (KAPA Biosystems), and sequenced as 50 bp single reads using an Illumina HiSeq instrument at the DNA Sequencing Core, University of Michigan. Relative read counts at gene level were estimated using HiSeq, and normalization and differential expression was performed with DESeq2 statistical package^33^. Genes were considered to be differentially expressed when the log2(fold change) ≥  ± 1.5, the adjusted *p* value was < 0.05, and the FDR was < 0.1%, in accordance with conventional thresholds. The differentially expressed (DE) genes were applied to iPathwayGuide (http://www.advaitabio.com) for functional classification analysis. All analysis was performed by the Bioinformatics Core, University of Michigan.

Clustering analyses were performed with a hierarchical method using an average linkage and euclidean distance metric to illustrate relationships among the data from complex array experiments using Cluster 3.0^34^. The clustering data were visualized using Java TreeView^35^.

### Statistical analysis

Data were analyzed using Prism 6 (GraphPad). All results were expressed as mean ± Standard Error of Mean (SEM). Statistical significance was determined by unpaired Student’s t-test and ANOVA analysis was performed for comparisons of three or more groups. A *p* value of less than or equal to 0.05 was considered statistically significant (*p < 0.05; **p < 0.01; and ***p < 0.00). All transgenics and wild-type controls having identical genetic background were used with sample size over 10 or otherwise stated.

## Results

### Generation of transgenic mice that specifically overexpressed miR-30d in β-cells

To fully understand the physiological function of miR-30d in β-cells in vivo, we generated and selected transgenic mice differentially overexpressing miR-30d under the transcriptional control of mouse insulin I gene promoter (MIP) (Fig. 1A). Seven independently derived founders were obtained by PCR genotyping. Compared to wild-type (WT) mice, the expression of miR-30d was significantly increased twofold in two lines and 15-fold in five lines of transgenic mice confirmed by quantitative real time PCR (RT-qPCR) (Fig. 1B). Of note, mice expressing MIP-GFP transgene were reported to secrete human growth hormone (hGH) due to inclusion of hGH gene^36^. However, no evidence indicated the activation of hGH-stimulated downstream signaling pathways in these transgenic mice despite the production of spliced hGH mRNA^37^. To overcome the potential limitation of the MIP-GFP mice, we selected two independent transgenic lines to perform the following experiments: one with low-level of transgenic overexpression (TgL, twofold) and the other with high-level of transgenic overexpression (TgH, 12-fold) (Fig. 1B). The genomic DNA was isolated for the determination of transgene copy numbers in these two lines. TgL mice contained 1 intact copy of the transgene whereas TgH mice carried 3 copies, as determined by qPCR (Fig. 1C). To keep the consistent transgene copy numbers in each line for the study, both TgL and TgH were continuously bred with wild-type mice, respectively. Thus, all transgenic mice used for the study were heterozygous. The aged-matched non-transgenic littermates were routinely used as WT control mice.

The expression of miR-30d showed no obvious difference in other tissues including brain, liver, heart, spleen or kidney in TgH when compared to WT which had identical genetic background (Fig. 1D). We further performed immunohistochemistry for GFP, the transgenic reporter, and insulin in the pancreas sections from transgenic and WT mice (Fig. 1E). The expression of the GFP reporter gene was restricted to insulin-producing β-cells of TgL and TgH mice. No GFP fluorescence was detected in WT mice. We also noted that only approximately 50–60% of β-cells co-expressed GFP in both TgH and TgL mice, which was consistent with previous reports that not all β-cells expressed GFP in MIP-GFP controlled transgenic mice^38, 39^. However, miR-30d expression showed an obvious induction (darker blue staining) in TgL and much more robust increase in TgH compared to WT mice, as determined by in situ hybridization of miR-30d (Fig. 1E). These data demonstrated successful generation of a mouse model with β-cell specific overexpressed miR-30d at low and high-levels.

### Transgenic miR-30d mice exhibited impaired glucose homeostasis under high-fat diet condition

**Figure 2:**
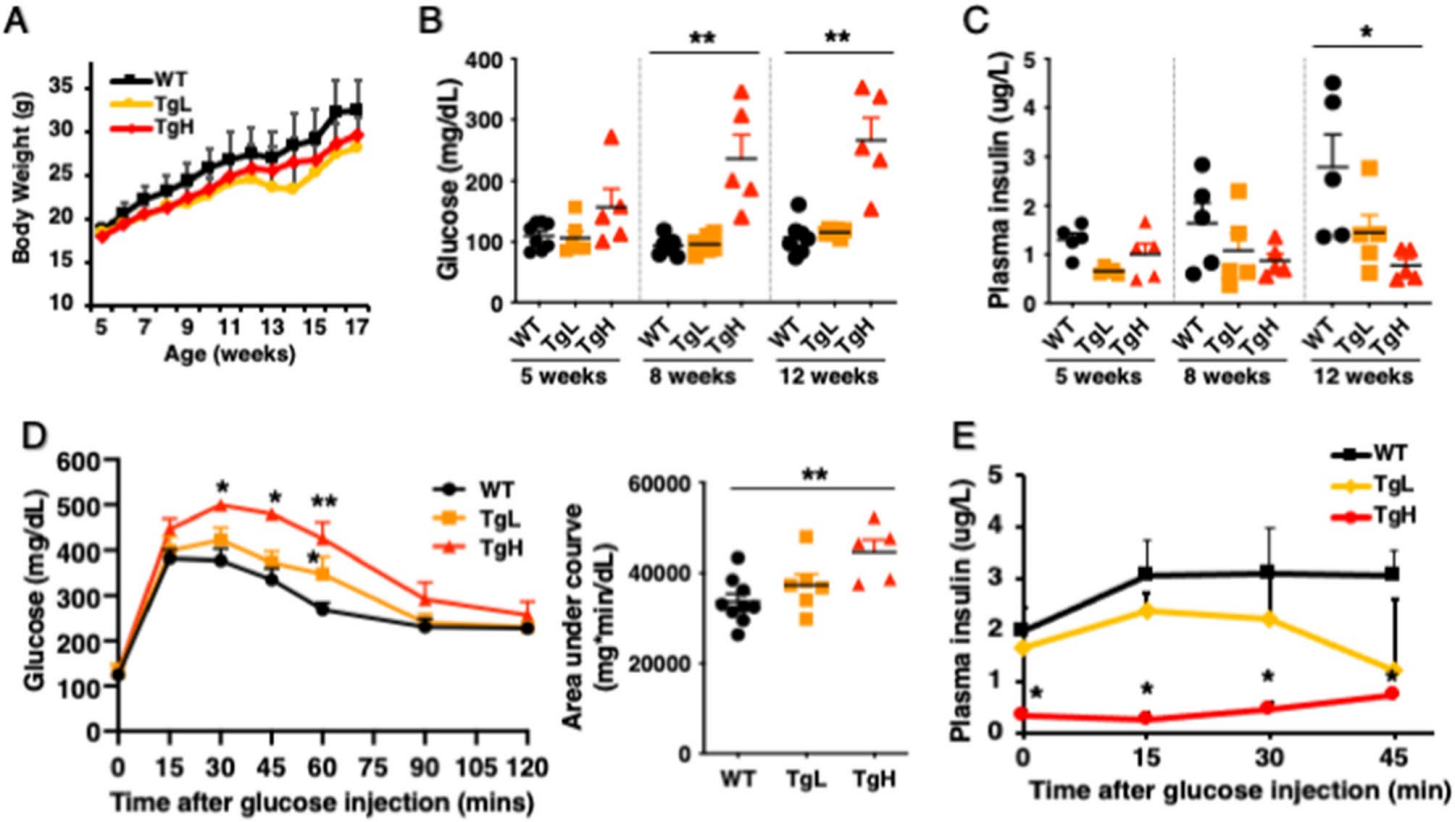
Transgenic overexpressed miR-30d impaired glucose homeostasis and reduced glucose tolerance in TgH compared to TgL and WT under high-fat diet treatment. (**A**) Body weight was measured weekly starting at 4-week-old when mice fed with high-fat diet (n = 4–6 per group). (**B**) Blood glucose was measured at weeks 5, 8 and 12 (n = 5 per group). (**C**) Plasma insulin was measured at weeks 5, 8 and 12 (n = 5 per group). (**D**) Glucose tolerance test was performed in male mice at 18-week-old. Mice were fasted for 16 h and intraperitoneally injected with glucose at 1.0 g/kg body weight. Blood glucose measurements were taken from the tail vein at 0, 15, 30, 45, 60, 90 and 120 min after injection. Area Under Curve was calculated as quantification of the glucose tolerance test curve (n = 5–8 per group). (**E**) Blood samples were collected simultaneously during the glucose tolerance test for plasma insulin ELISA (n = 4–6 per group). All data were presented as mean ± SEM. *p < 0.05, **p < 0.01 versus WT.

No obvious changes were observed in blood glucose and plasma insulin among WT, TgL and TgH when mice were fed normal diets. The glucose tolerances had no obvious difference in TgL and TgH mice when compared to WT mice at either 4-week-old or 14-week-old (Supplemental Figure 1). Evidence has indicated that miRNAs are stress regulators, and the metabolic phenotype might be subtle in transgenic mice fed normal diet^22, 40^. Therefore, we explored the metabolic phenotype in transgenic mice fed HFD. Compared to HFD-fed WT, both TgL and TgH mice gained similar body weight during the 5–17 weeks of HFD feeding (Fig. 2A). The blood glucose started to rise dramatically in TgH starting from 8-week-old compared to WT mice (Fig. 2B). TgL mice however maintained comparable glucose level as WT at 5-, 8- and 12-week-old. Plasma insulin was further determined, and a steady increase was shown in WT as the insulin resistance developed under HFD treatment (Fig. 2C). In contrast, TgH mice had lower steady state plasma insulin levels, which may lead to HFD-induced hyperglycemia in TgH mice.

Glucose tolerance test was performed on male mice after 16 weeks of HFD feeding (Fig. 2D). While wild type mice still could restore normal blood glucose 2 h after the glucose injection, the blood glucose in TgH mice was much higher during the two hours after glucose administration (e.g. above 400 mg/dl observed 1 h after glucose administration), indicating that TgH displayed significant glucose intolerance compared to WT mice. Moreover, TgH mice had much lower plasma insulin levels during the whole glucose challenge test (Fig. 2E). In contrast, TgL mice obtained regular glucose response and had comparable insulin released in plasma as WT mice. Taken together, the results indicated that high-level expression of miR-30d promoted HFD-induced hyperglycemia and glucose intolerance.

### Overexpression of miR-30d induced β-cell apoptosis in response to high-fat diet

**Figure 3:**
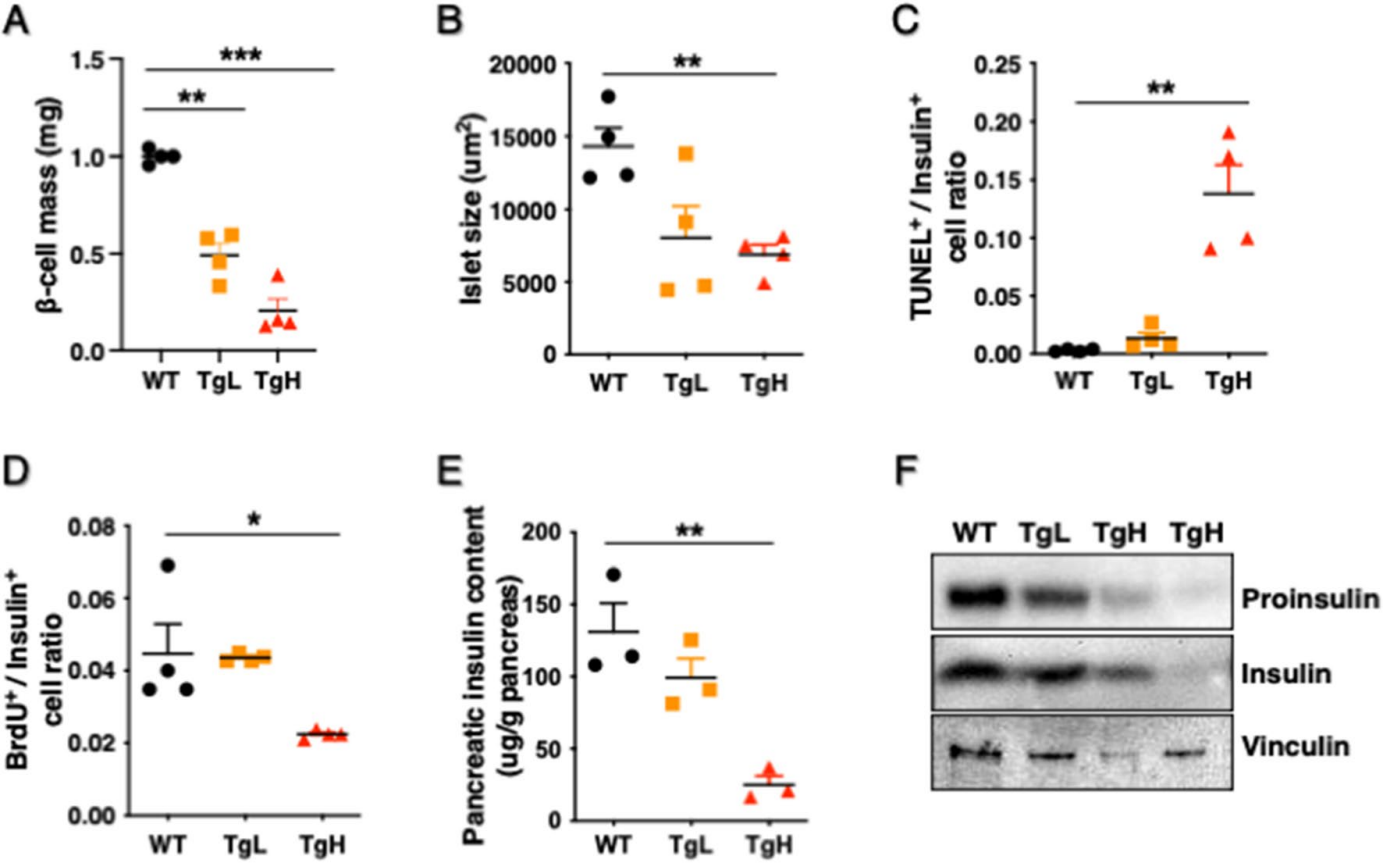
Overexpression of miR-30d significantly induced β-cell apoptosis in TgH mice. **(A)** Pancreas sections were immunostained with anti-insulin. The islet area and the area of each section was determined with the ImagePro image software. β-cell mass (mg) was calculated by multiplying the ratio of insulin-positive areas by pancreatic weight (n = 4 per group). (**B**) Islet size was calculated by dividing the area of insulin-positive islets by the total islet numbers (n = 4 per group). (**C**) Sections were performed in situ TUNEL assay, followed by staining with anti-insulin and DAPI. The percentage of TUNEL-positive β-cells was calculated and divided by total number of insulin positive cells. At least 2000 β-cell nuclei were counted per pancreas (n = 4 per group). (**D**) BrdU (100 μg/g body weight) was administrated for 7 consecutive days through i.p. injection in HFD mice at 20-week-old. Pancreas sections were stained with anti-BrdU and subsequently with anti-insulin. The percentage of BrdU-positive β-cells was calculated and divided by total number of insulin-positive cells. At least 2000 β-cell nuclei were counted per pancreas (n = 4 per group). (**E**) Whole pancreas tissues were collected from mice at 20-week-old and homogenized in acid ethanol. Insulin content was measured and normalized to the pancreas weight (n = 3 per group). (**F**) Western blots confirmed that both proinsulin and mature insulin protein levels were reduced in islets isolated from TgH mice compared to TgL and WT mice. Islets were lysed and analyzed by immunoblotting with anti-proinsulin, anti-insulin, and anti-Vinculin (loading control). All data were presented as mean ± SEM. *p < 0.05, **p < 0.01, or ***p < 0.001 versus WT.

To examine why TgH mice exhibited hyperglycemia and lower plasma insulin levels, pancreas sections were prepared, and β-cell mass and islet size were analyzed by immunostaining for insulin. Compared to the large expansion of β-cell mass in WT in response to HFD, TgH showed a significant decrease in β-cell mass and size (Fig. 3A, B). β-cell apoptosis and proliferation were further examined in order to understand what triggered the loss of β-cell mass in TgH. Apoptotic cells were detected by TUNEL-positive nuclei and the proportion of TUNEL-positive β-cells was significantly increased in TgH compared to TgL and WT (Fig. 3C). Increased apoptosis was also detected by increased cleaved caspase 3-positive β-cells in TgH (data not shown). In contrast, quantification of BrdU positive β-cells, a DNA synthesis-based cell proliferation assay, showed a minor decrease in TgH compared to WT and TgL (Fig. 3D). The results indicated that highly overexpressed miR-30d remarkably induced β-cell apoptosis with no compensatory growth of β-cells in TgH mice under HFD challenge. However, less than 2 times of overexpression of miR-30d in TgL had no significant effects on β-cell apoptosis when compared to WT mice.

Consistently, the insulin content in the whole pancreas was significantly lower in TgH than WT (Fig. 3E). Western blot confirmed that both proinsulin and mature insulin protein levels were largely decreased in isolated islets of TgH compared to WT and TgL (Fig. 3F).

### MiR-30d overexpression increased glucagon content and insulin/glucagon bihormonal cells in mice fed with high-fat diet

**Figure 4:**
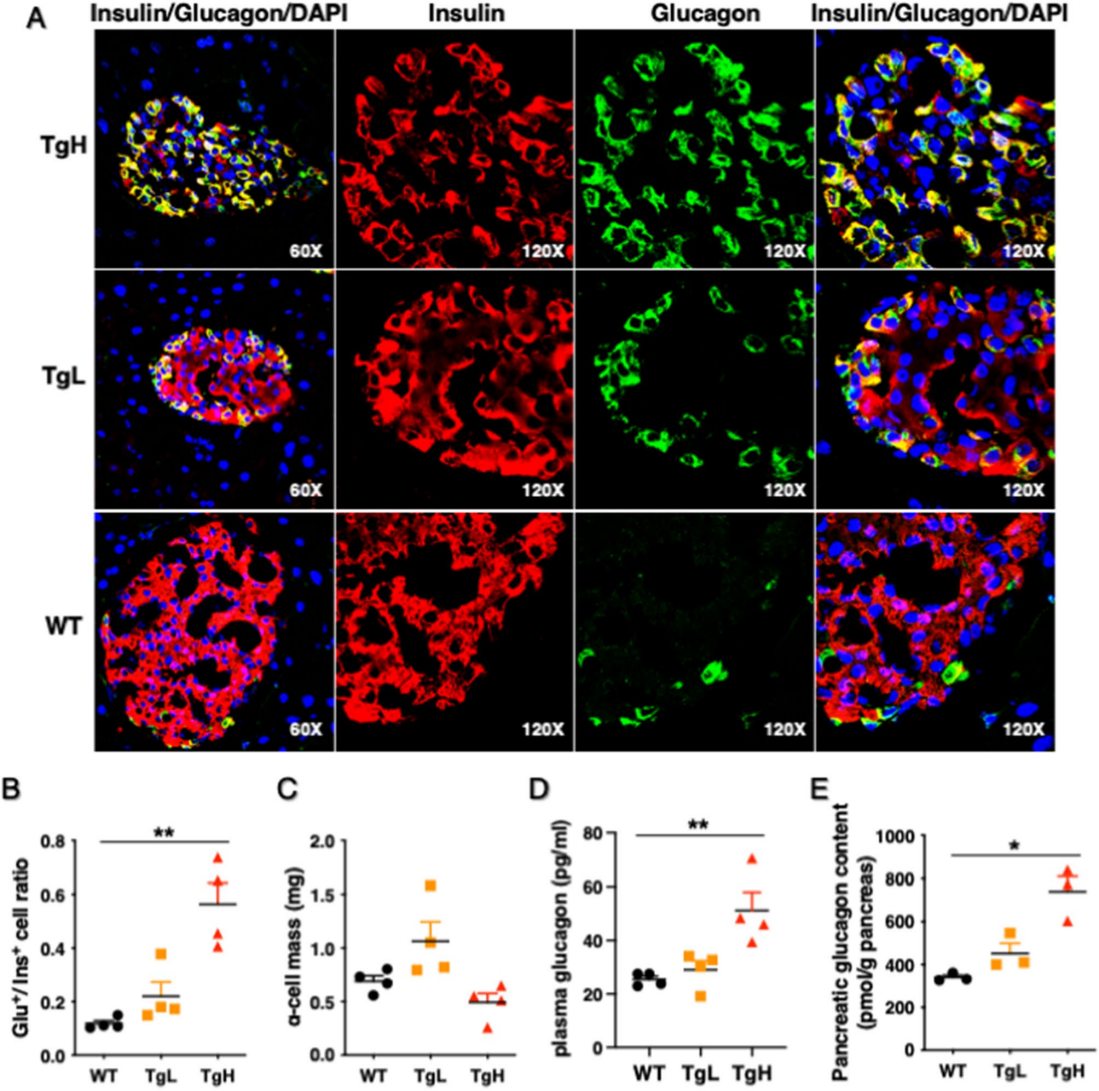
MiR-30d overexpression increased plasma glucagon, glucagon content and bihormonal cells (insulin^+^/glucagon^+^ cells) in mice fed high-fat diet. **(A)** Pancreas sections were co-stained with anti-insulin (red) and anti-glucagon (green) antibodies and visualized by a confocal immunofluorescence microscopy. Nuclei were stained with DAPI (blue). The three-color overlay images showed bihormonal cells (indicated by double-positive yellow cells) in TgH and TgL. Magnification: 60× or 120×. (**B**) The α/β-ratio was calculated by dividing the number of glucagon-positive α-cells by the number of insulin-positive β-cells (n = 4 per group). (**C**) α-cell mass (mg) was calculated by multiplying the ratio of glucagon-positive areas by pancreatic weight (n = 4 per group). (**D**) Plasma glucagon levels measured at 12-week-old (n = 4 per group). (**E**) Whole pancreas tissues were collected and homogenized in acid ethanol. Glucagon content was measured and normalized to the pancreas weight (n = 3 per group). All data were presented as mean ± SEM. *p < 0.05 or **p < 0.01 versus WT.

Due to hyperglycemia and reduced insulin content in TgH mice, we examined α-cell distribution and glucagon contents in HFD-fed mice at 18-week-old. Confocal imaging of sections revealed the presence of insulin-positive (insulin^+^) cells that also expressed glucagon (i.e., bihormonal cells in yellow) in transgenic mice (Fig. 4A). Interestingly, these bihormonal cells were present at a markedly increased frequency in mice fed HFD compared to those fed normal diet (ND) (Supplemental Figure 2). Quantitative analysis revealed that the relative areas of α- and β-cells (α/β-ratio) were significantly higher in TgH compared with WT although α-cell mass was not significant different between them (Fig. 4B, C). Consistently, plasma glucagon and total glucagon content were increased almost twofold in TgH compared to WT (Fig. 4D, E). Of note, although TgL did not produce statistically significant changes in α/β-ratio and glucagon content, bihormonal cells started to be observed in TgL (Fig. 4A). These data suggest that miR-30d overexpression might induce β-cell dedifferentiation by converting β-cells to α-like-cells, which is consistent with the loss of β-cell and gain of α-cell characteristics in individuals with type 2 diabetes^5, 41^.

### miR-30d overexpression triggered downregulation of β-cell-enriched genes and upregulation of α-cell-enriched genes

**Figure 5:**
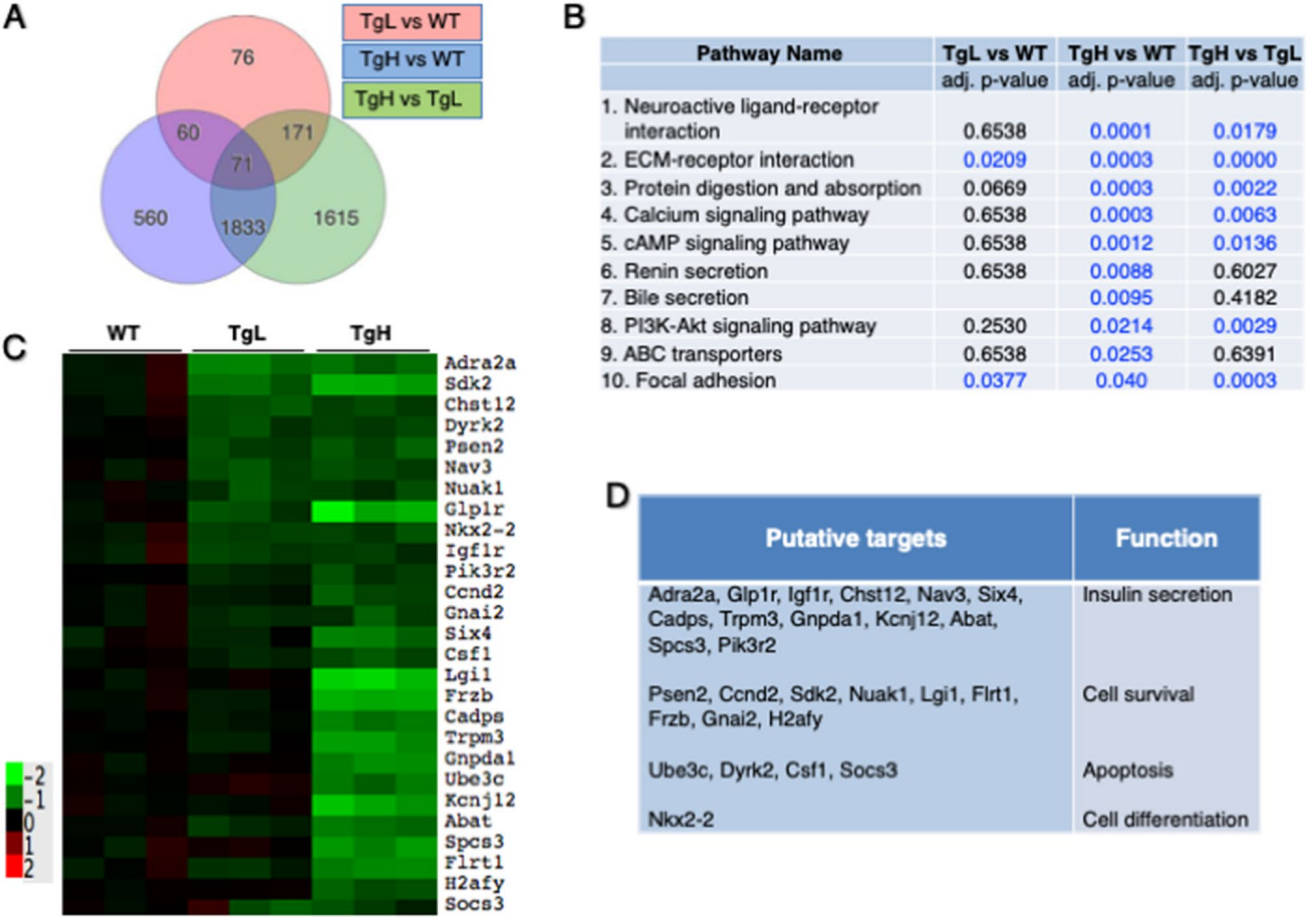
RNA sequencing revealed differentially expressed (DE) genes in transgenic and control mice. Differentially expressed genes (false discovery rate < 0.01) between WT, TgL and TgH mice (n = 3 mice in each group) were generated by RNA-sequencing using Illumina HiSeq 4000. (**A)** Proportional Venn diagram representing the number of DE genes in TgL, TgH or both (overlap). (**B)** Gene ontology analysis revealed top 10 significantly enriched “biological pathways”. Data were analyzed by Fisher/binominal test with Bonferroni-adjusted *p* value. The numbers highlighted in blue represent the designated significance threshold (*p < 0.05). (**C**) A heatmap of expression levels of putative target genes of miR-30d identified by intersecting DE genes with miRNA TargetScan databases. Color scale represents their expression across groups. (**D**) List of selected target genes and their function.

To evaluate the transcriptomic difference between transgenic and control mice, RNAseq analysis was performed with islets isolated from WT, TgL and TgH mice, and analyzed for differentially expressed (DE) genes using DESeq2 method. As compared to WT mice, 378 genes in TgL and 2524 genes in TgH were differentially expressed (Fig. 5A, Supplemental Table 1). Of these, 131 DE genes were differentially expressed in both TgL and TgH. To study the biological relevance and consequences of the DE genes in transgenic mice, KEGG pathway analysis was conducted and revealed that most of DE genes in TgH were involved in neuroactive ligand-receptor interaction, insulin-regulated calcium signaling and PI3K-Akt signaling pathways (Fig. 5B). The pathways over-represented in both TgL and TgH were extracellular matrix (ECM)-receptor interaction and focal adhension (Fig. 5B).

Since miRNAs primarily act by binding to the 3′UTR of their target mRNAs thereby promoting mRNA degradation, we explored if any of these DE genes are predictable targets of miR-30d. After intersecting downregulated DE genes with the TargetScan database, which include miRNA predicted targets, we identified 38 and 99 potential targets downregulated in TgL and TgH mice, respectively. Hierarchical cluster analysis indicated that some candidate targets were more effectively repressed in TgH compared to TgL (Fig. 5C, Supplemental Table 2). 27 putative targets were selected to be either experimentally validated or play important function in β-cells, such as Nkx2.2 and Socs3 (Fig. 5D).

**Figure 6:**
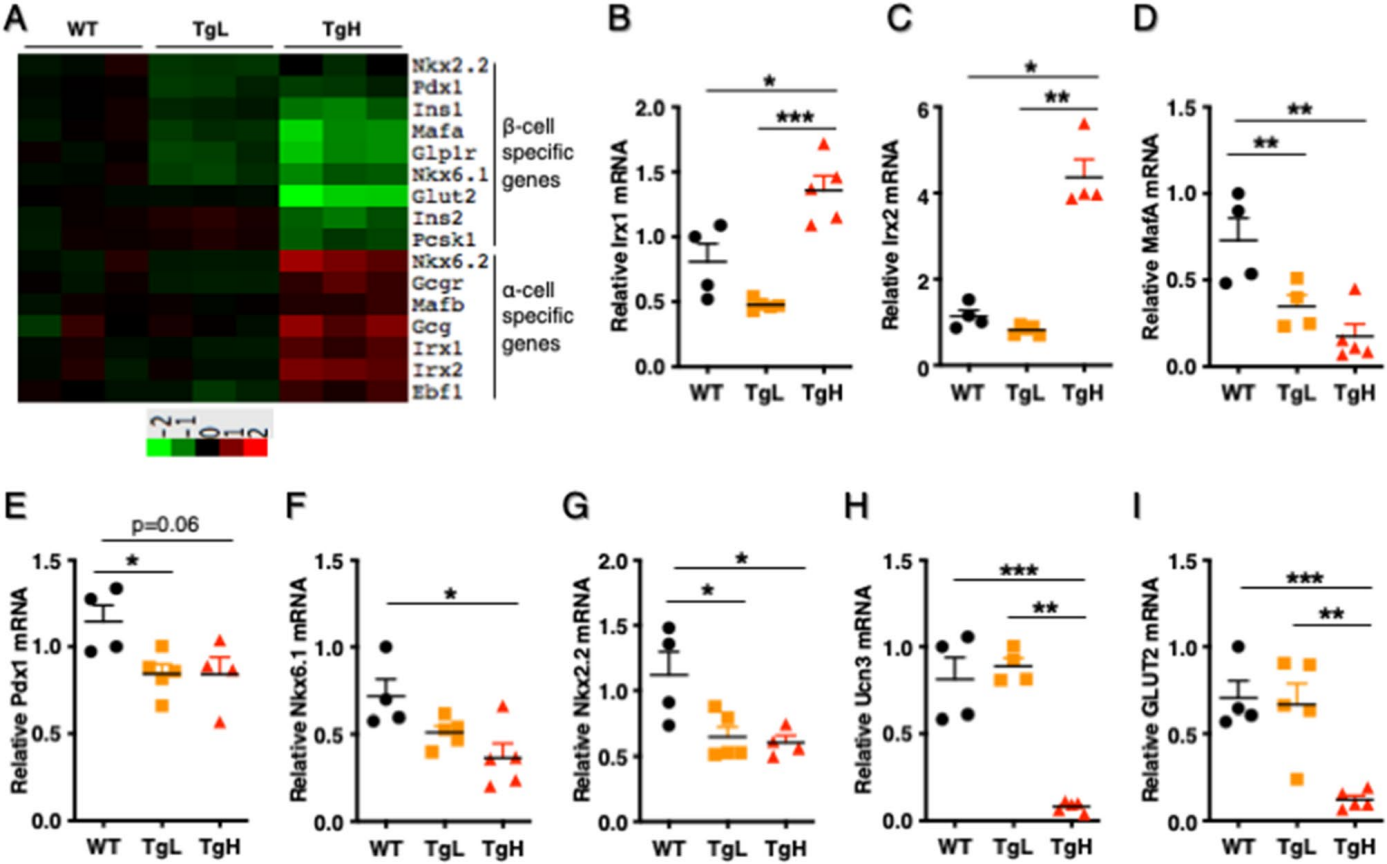
Transgenic overexpressed miR-30d induced downregulation of β-cell-enriched genes and upregulation of α-cell-enriched genes in TgH mice. **(A)** A heatmap of expression levels of major β-cell and α-cell-enriched genes revealed from RNAseq. (**B–I)** RT-qPCR validated the relative mRNA expression of key genes (n = 4–5 per group). All data were presented as mean ± SEM. *p < 0.05; **p < 0.01; or ***p < 0.001 versus WT.

In addition, RNAseq analysis revealed distinct expression changes in a group of β-cell and α-cell enriched genes (Fig. 6A, Supplemental Table 3). To validate these changes, RT-qPCR was performed to measure gene expression in islets isolated from three groups of mice. As expected, we observed significant downregulation in β-cell enriched genes including Ins1, Ins2, β-cell specific transcription factors MafA, Nkx6.1, Nkx2.2, Ucn3 and glucose transporter Glut2 in TgH (Fig. 6D–I). In contrast, the expression of α-cell enriched genes (Irx1 and Irx2) were highly increased in TgH mice when compared to TgL and WT (Fig. 6B, C). Among these validated markers, only MafA, Pdx1 and Nkx2.2 showed relative reduction in TgL mice (Fig. 6D, E, G). These results agreed with the observation that TgH mice exhibited a greater reduction of insulin content and a more enrichment of glucagon content.

## Discussion

Studies have showed that expression of miR-30d was downregulated in islets of HFD-induced obese mice^42^ and diabetic *db/db* mice^25^. Here, we found that transgenic overexpression of miR-30d in β-cells systemically affected β-cell function in an expression-dependent manner: low-level of miR-30d (TgL) had mild effects on signaling pathways and displayed no significant phenotypic changes, whereas high-level of expression (TgH) impaired important signaling pathways and led to diet-induced hyperglycemia and β-cell dysfunction. In addition to reduced insulin content, noteworthy is the presence of insulin^+^ cells that also expressed glucagon (i.e., bihormonal cells), which stimulated large increases in plasma glucagon levels in TgH mice. Taken together, these results point to the importance of an appropriate level of miR-30d in maintaining mature β-cell identity.

MiRNAs are required to be expressed within optimal levels in the cell, i.e., too high or too low expression would be detrimental for cellular functions^43^. Various studies have shown that under normal condition many miRNA gain- or loss-of-functions have subtle effects, which can become more pronounced when the organism is adversely challenged^21, 44, 45^. We found no major physiological changes displayed in TgL mice, possibly due to the presence of redundant mechanisms selectively activated upon adaptation of the β cell to low-level expression of miR-30d in TgL mice. In contrast, as miR-30d level increased, a grossly perturbed glucose homeostasis was shown in TgH, in which many important pathways were altered, including neuroactive ligand-receptor interaction, insulin-regulated calcium signaling and PI3K-Akt signaling pathways. Impaired regulation of these pathways is believed to interfere with β-cell function and result in severe defects in glucose metabolism. In addition, the altered ECM-receptor interaction pathway and its involved DE genes require for further characterization. All these observations provide a possible mechanism for the finetuning of β-cell function by miR-30d.

Mechanistically, each miRNA has the ability to repress hundreds of mRNA targets, but a miRNA most likely targets only a small fraction of targets under specific tissues or physiological conditions^46, 47^. Previous studies have identified that overexpressed miR-30d induced insulin production in isolated islets and MIN6 cells by targeting mitogen-activated protein 4 kinase 4 (Map4k4)^24, 25, 48^. More recently, transfection of miR-30d mimic in isolated islets was reported to inhibit apoptosis in isolated islets by inhibiting suppressors of cytokine signaling 3 (Socs3)^49^. However, the systematic manipulation of miR-30d in islets reduced insulin contents and increased apoptosis. These inconsistent effects in β-cells suggest a significant difference between the transient modification of miR-30d in isolated individual cells (in vitro) and the systematic overexpression of miR-30d in interacting cells of transgenic islets (in vivo). The systematic manipulation of miR-30d in islets can trigger coordinated changes of multiple targets (such as Nkx2.2, Glp1r and Igf1r) to allow sustained β-cell function, and extra overexpression of miR-30d may fail to balance and promote β-cell failure. The expression level of miR-30d was correlated with its repressive activity, and high-level of miR-30d repressed its target mRNAs more. Moreover, miR-30 family includes five members and the manipulation of one of them might be compensated by changes in the expression of other members^24^. Thus, it is not surprising that RNA sequencing revealed a more complex and widespread network of genes influenced by overexpression of miR-30d in transgenic mice than previously reported in vitro systems. Impacts of miR-30d on other related members and the combinational modification of multiple targets require further investigation.

In addition to increased apoptosis, transgenic overexpression of miR-30d also induced β-to-α-cell conversion based on the appearance of bihormonal cells and excess plasma glucagon levels. Consistently, a group of β-cell-enriched genes (such as MafA, Pdx1 and Nkx6.1) was repressed whereas several α-cell-enriched genes (such as Irx1 and Irx2) were activated in response to high-level expression of miR-30d. Nkx2.2, one of miR-30d potential targets, is a transcription factor required for β-cell differentiation and mutation of Nkx2.2 leads to β-to-α-cell conversion^50^. Whether miR-30d plays a role in β-to-α-cell conversion via Nkx2.2 remains to be examined. However, β-to-α-cell conversion may be directly contributed by chronic hyperglycemia displayed in TgH mice. Hyperglycemia exerts a strong alterations in gene expression, which contributes to loss of β-cell identity and function in diabetes^51, 52^.

Collectively, the present study dissected systemically metabolic effects of miR-30d in transgenic mice. Our results indicated that an appropriate miR-30d level is important in maintaining β-cell identity. Considering that most miRNAs are conserved between human and mouse, the use of mouse transgenic or knockout models is warranted to be useful in evaluating individual miRNA regulatory networks in vivo. The study also provides insight that miRNA levels must be maintained within a given range to have a therapeutic effect.

## Supplementary Information

Supplementary Information 1.

Supplementary Information 2.

Supplementary Information 3.

Supplementary Information 4.
