# Supplementary material for: Transgenic overexpression of microRNA-30d in pancreatic beta-cells progressively regulates beta-cell function and identity
Source: Sci Rep. 2022 Jul 13;12:11969. doi: 10.1038/s41598-022-16174-7 (PMC9279310; doi:10.1038/s41598-022-16174-7)
Supplement: Supplementary file 4 — Supplementary Information 4. [file 41598_2022_16174_MOESM4_ESM.docx]

**Supplementary Figures and Tables**


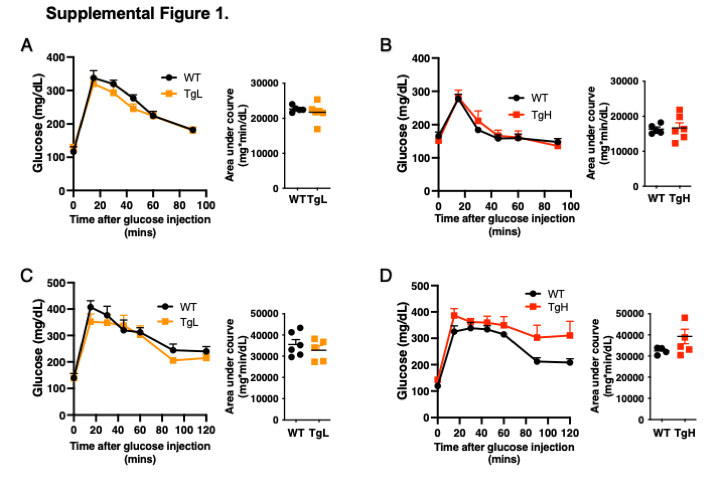


**Supplemental Figure 1. Elevated miR-30d expression had no significant effects on glucose tolerance in mice fed with normal diet.** **A, B.** Glucose tolerance test was performed in TgL (A) or TgH (B) at 4-week-old. Mice were fasted for 16 hrs and intraperitoneally injected with glucose at 1.5 g/kg body weight. Blood glucose measurements were taken from the tail vein at 0, 15, 30, 45, 60, 90 and 120 min after injection. Area Under Curve was calculated as quantification of the glucose tolerance test curve (n=4-6 per group). **C, D.** Glucose tolerance test was assessed in TgL vs. WT (C) or TgH vs WT (D) at 14-week-old (n=4-6 per group).


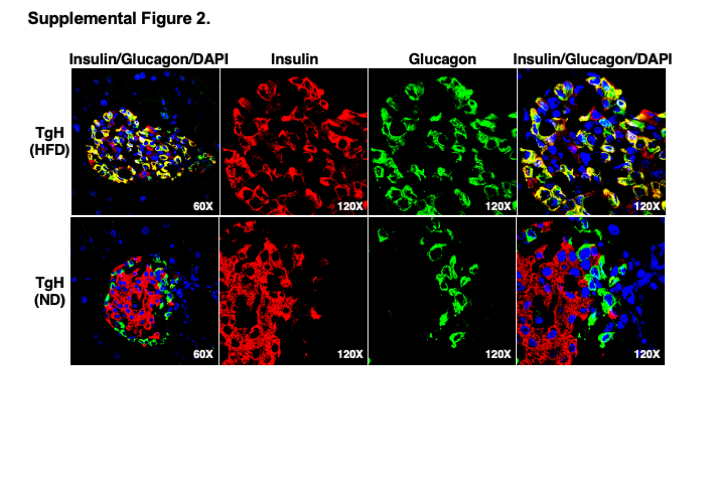


**Supplemental Figure 2.** **Bihormonal cells (insulin^+^glucagon^+^ cells) were increased in TgH mice fed with high-fat diet (HFD) compared that fed with normal diet (ND).** Pancreatic sections were prepared and co-stained with anti-insulin (red) and anti-glucagon (green) antibodies. Nuclei were stained with DAPI (blue). Note: Yellow showed double-positive (Ins^+^Gcg^+^) cells.


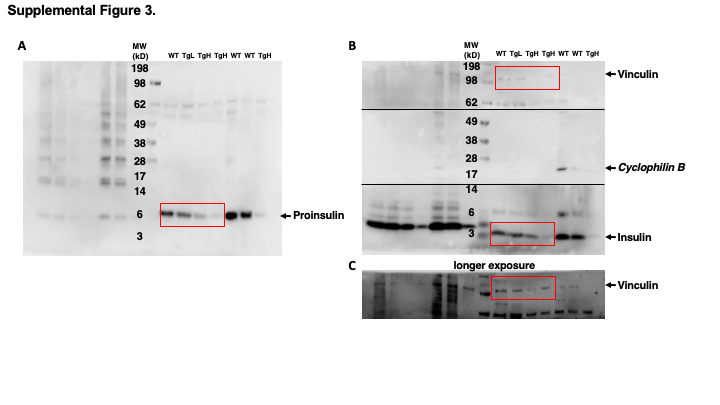


**Supplemental Figure 3.** Original western blot images of Figure 3F. Isolated islets were lysed in RIPA buffer, divided into two portions and resolved on 4–12% Bis-Tris NuPAGE gels (Invitrogen). One blot was immunoblotted with anti-proinsulin (A). The other blot was cut into three sections for anti-insulin (B, bottom), anti-Cyclophilin B (B, middle) and anti-Vinculin (B, top), respectively. The anti-Vinculin blot was exposed much longer and showed separately (C). Red boxes represent the regions showed in the Figure 3F. The positions of molecular mass markers are noted.

**Supplementary Table Legends**

**Supplemental Table 1.** List of top 150 differentially expressed genes revealed from RNAseq.

**Supplemental Table 2.** List of miR-30d putative target genes identified by intersecting differentially expressed genes with miRNA target databases.

**Supplemental Table 3.** List of major α-cell and β-cell-enriched genes with differential expression revealed from RNAseq.
